# The influence of mosquito resting behaviour and associated microclimate for malaria risk

**DOI:** 10.1186/1475-2875-10-183

**Published:** 2011-07-07

**Authors:** Krijn P Paaijmans, Matthew B Thomas

**Affiliations:** 1 Center for Infectious Disease Dynamics & Department of Entomology, Merkle Lab, Penn State University, University Park, PA 16802, USA

## Abstract

**Background:**

The majority of the mosquito and parasite life-history traits that combine to determine malaria transmission intensity are temperature sensitive. In most cases, the process-based models used to estimate malaria risk and inform control and prevention strategies utilize measures of mean outdoor temperature. Evidence suggests, however, that certain malaria vectors can spend large parts of their adult life resting indoors.

**Presentation of hypothesis:**

If significant proportions of mosquitoes are resting indoors and indoor conditions differ markedly from ambient conditions, simple use of outdoor temperatures will not provide reliable estimates of malaria transmission intensity. To date, few studies have quantified the differential effects of indoor *vs *outdoor temperatures explicitly, reflecting a lack of proper understanding of mosquito resting behaviour and associated microclimate.

**Testing the hypothesis:**

Published records from 8 village sites in East Africa revealed temperatures to be warmer indoors than outdoors and to generally show less daily variation. Exploring the effects of these temperatures on malaria parasite development rate suggested indoor-resting mosquitoes could transmit malaria between 0.3 and 22.5 days earlier than outdoor-resting mosquitoes. These differences translate to increases in transmission risk ranging from 5 to approaching 3,000%, relative to predictions based on outdoor temperatures. The pattern appears robust for low- and highland areas, with differences increasing with altitude.

**Implications of the hypothesis:**

Differences in indoor *vs *outdoor environments lead to large differences in the limits and the intensity of malaria transmission. This finding highlights a need to better understand mosquito resting behaviour and the associated microclimate, and to broaden assessments of transmission ecology and risk to consider the potentially important role of endophily.

## Background

The transmission intensity of malaria is strongly influenced by environmental temperature [1-5]. This effect derives from the fact that malaria mosquitoes are small, coldblooded insects, with body temperatures that will tend to closely track that of the direct surrounding environment. As temperature changes, so will mosquito (and parasite) physiology and associated ecology.

Most biological process-based models that approximate malaria risk use mean (often monthly) outdoor air temperature to estimate the various mosquito and parasite life history parameters that combine to determine transmission intensity [1,6-11]. This approach has been challenged recently with studies demonstrating, for example, the additional influence of daily temperature variation [5,12] and the importance of microclimatic differences between adult and larval mosquito habitats [13]. The aim of the current study is to highlight a further factor shaping the effects of temperature on malaria risk, namely the influence of indoor *vs *outdoor temperatures.

The mosquito gonotrophic cycle (blood-feeding, egg maturation and oviposition, which are repeated several times throughout adult life) can be as short as two days, but could take over a week, depending on temperature [14-16]. If a mosquito lays eggs and searches for a new blood meal during a single night (as appears appropriate for *Anopheles gambiae*, which has been observed to deposit the majority of eggs in the first hours after sunset [17,18] and to bite throughout the night [19-21]), then a large portion of the gonotrophic cycle is spent resting. But where do mosquitoes rest?

**Figure 1:**
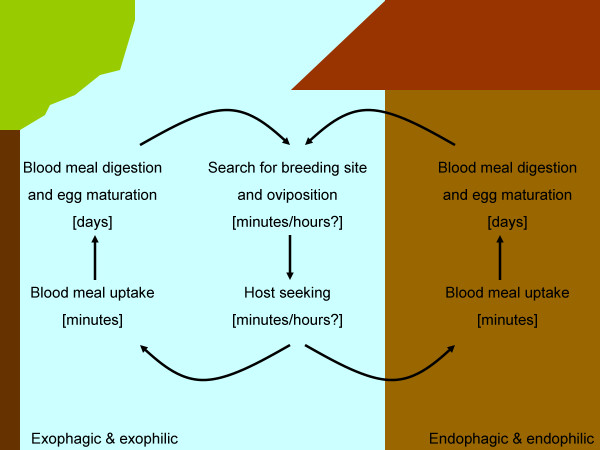
**Schematic overview of the gonotrophic cycle of mosquitoes that both feed (exophagic) and rest (exophilic) outdoors (left hand side of figure), or both feed (endophagic) and rest (endophilic) indoors (right hand side of figure)**. At these extremes, mosquitoes will spend the majority of their adult lives under dramatically different environmental conditions.

Mosquito resting behaviour can be divided into two categories. An endophilic mosquito is defined as a mosquito that rests indoors, inside a human dwelling, during the period between the end of blood-feeding and the onset of searching for an oviposition site [22]. An exophilic mosquito spends this period somewhere outside the human dwelling (Figure 1). Of the key malaria vectors in sub-Saharan Africa, *Anopheles gambiae *s.s. is thought to be largely endophilic and spend considerable time indoors [23-27], although exophily has been reported [28,29]. *Anopheles funestus *is also usually classified as a more endophilic species [25,26,30-33] but again, exophilic behaviour is observed [34]. *Anopheles arabiensis*, in contrast, is classified as a more exophilic species [23,25,29,33-36], although the reverse behaviour (endophily) is also reported [24,27,37,38].

Overall, while there appear some generalizations, resting behaviour appears to be relatively plastic with considerable potential for variation between and within species. Indeed, a couple of studies have reported no significant tendency to repeated endophily or exophily for even the same individual mosquito [26,39].

## Presentation of the hypothesis

If mosquitoes are at least partially endophilic, then estimating transmission intensity requires measures of indoor as well as outdoor temperatures. Unfortunately, there are only a few studies that actually measure the mean indoor and outdoor temperature simultaneously, and even fewer studies that keep track of the daily temperature variability. From the limited studies that exist, it appears that indoor temperatures in traditional houses are a few degrees Celsius higher than the outdoor temperature (Table 1, [14,40-44]). Additionally, the indoor daily temperature range (or DTR, the difference between daily minimum and maximum temperature) in traditional houses tends to be smaller than the outdoor DTR (Table 1, [45,46]). That said, and the relatively modest DTRs shown in Table 1 notwithstanding, large DTRs of 10-15°C are commonly observed indoors [40,41,47,48].

**Table 1:** Outdoor and indoor microclimatic data (mean temperatures and temperature variability) in several villages at various altitudes in Tanzania and Kenya

				Outdoor temperature				Indoor temperature			
**Year**	**Country**	**Village (#)**	**Altitude(m)**	**mean**	**min**	**max**	**DTR**	**mean**	**min**	**max**	**DTR**

1996	Tanzania	Kwameta (1)	335	24.7	20.2	30.4	10.2	25	21.9	28.8	6.9
		Magundi (2)	640	22.7	19.6	27.4	7.8	25.7	23.7	28.4	4.7
		Kwamhanya (3)	775	22	18.9	26.6	7.7	25.8	23	29.2	6.2
		Bagamoyo (4)	1040	19.4	16	23.7	7.7	22.1	20	24.7	4.7
		Balangai (5)	1360	17.8	15.2	21.8	6.6	19.6	18.1	21.2	3.1
		Milungui (6)	1686	15.7	11.7	20.6	8.9	19.5	18.2	20.6	2.4

2004	Kenya	Kombewa (7)	~1200	22.5	17.6	29.7	12.1	23.1	20.1	26.5	6.4
		Marani (8)	1500-1650	19.5	14.4	27.4	13	21.5	19.2	24.7	5.5

References: Village 1-6 [50], villages 7&8 [54].

The difference between indoor and outdoor temperatures is expected to alter temperature-related estimates of transmission intensity. Some studies have attempted to account for this effect by applying a simple temperature correction [49-51], and one study included actual indoor temperature [41]. However, few (if any) studies have quantified the differential effects of indoor *vs *outdoor temperatures explicitly.

## Testing of the hypothesis

The parasite development time, or extrinsic incubation period (EIP) is one of the most influential parameters determining transmission intensity, as defined by the basic reproductive number, *R_0 _*[52]. EIP is known to be highly temperature sensitive [5,52,53]. Based on the data presented in Table 1 the effects of mean temperature and DTR inside and outside human dwellings on EIP was assessed using two different malaria development models: the widely used day-degree Detinova model [53], and the non-linear thermodynamic model proposed by Paaijmans and colleagues [12]. Using the reported minimum and maximum temperatures (Table 1, [50,54]), air temperature [55] and EIP [for methods see 12] were modelled at 30min intervals.

Table 2a shows that small differences in temperature between indoor and outdoor environments can have a large impact on the estimated length of the EIP. With warmer indoor temperatures, parasite development is faster than predicted from ambient conditions, with differences tending to becoming larger at higher altitudes as indoor and outdoor temperatures become more divergent. At the extreme, indoor environmental temperatures can enable parasites to complete incubation at altitudes where outdoor temperatures fall below the threshold for development. Thus, the environmental limits for malaria transmission depend not just on ambient conditions, but also on indoor conditions and the extent of endophily. These patterns are qualitatively similar for both temperature-development models. The patterns also hold up when the influence of diurnal temperature fluctuations is included, although temperature variation makes the differences in growth between indoor and outdoor environments slightly less marked.

**Table 2:** Parasite development time (EIP) and relative change in malaria risk (R_0_) based on indoor and outdoor temperatures

Village #	(a) Parasite development time (days)									(b) Percent change in *R_0_*for indoor environments relative to outdoor		
	**Mean temperatures**						**Fluctuating temperatures**			**Mean temperatures**		**Fluctuating temperatures**

	**Detinova**			**Paaijmans**			**Paaijmans**			**Detinova**	**Paaijmans**	**Paaijmans**
	
	**EIP_out_**	**EIP_in_**	**EIP_in-out_**	**EIP_out_**	**EIP_in_**	**EIP_in-out_**	**EIP_out_**	**EIP_in_**	**EIP_in-out_**			

1	12.8	12.3	-0.4	12.1	11.7	-0.3	12.9	12.1	-0.8	+7	+5	+12
2	16.6	11.4	-5.1	15.3	11.0	-4.3	15.0	11.1	-3.8	+117	+91	+78
3	18.5	11.3	-7.2	17.0	11.0	-6.1	16.5	11.3	-5.2	+195	+149	+119
4	32.6	18.2	-14.5	29.0	16.7	-12.3	28.5	16.9	-11.6	+784	+536	+478
5	ND^†^	30.8	N/A	50.1	27.5	-22.5	42.1	28.4	-13.7	N/A	+2889	+693
6	ND^††^	31.7	N/A	ND^†^	28.3	N/A	ND^†^	30.1	N/A	N/A	N/A	N/A
7	17.1	15.6	-1.4	15.8	14.5	-1.3	15.8	15.1	-0.7	+24	+21	+12
8	31.7	20.2	-11.5	28.3	18.5	-9.8	23.6	18.2	-5.4	+469	+337	+126

Parasite development was calculated with two different published models [Paaijmans' equation ref. 12, Detinova's equation ref. 53] using the outdoor and indoor temperature data presented in Table 1. EIP_out _indicates parasite development derived from outdoor temperatures, EIP_in _indicates development derived from indoor temperatures and EIP_in-out _indicates the number of days difference in parasite development indoors compared with outdoors.

^† ^No development: completion of parasites development takes longer than the upper limit for mosquito survival of 56 days [1]

^†† ^No development: temperature below lower threshold for *P. falciparum *development.

The EIP is just one of a range of parameters used to determine *R_0 _*[52]. Assessing the absolute effects of indoor *vs *outdoor temperatures on *R_0 _*requires estimates of all parameters. However, by taking the simplifying (and conservative) assumption that other parameters remain constant, the relative consequences of changes in parasite development rate on *R_0 _*can be assessed using the relationship between EIP and median daily mosquito survival rate, *p*, derived from the *R_0 _*equation: *p*^EIP^/-ln*p *[see 12 for more details]. Importantly, adult mosquito survival is largely insensitive to temperature across much of the transmission range, with mortality only increasing markedly as mean temperature exceeds 35-36 °C [1]. Keeping *p *fixed (in this case a median daily survivorship of *p *= 0.86, as used elsewhere [56]) enables the relative change in *R_0 _*due to effects of indoor *vs.* outdoor temperature on EIP to be estimated.

As expected, the shorter EIPs resulting from the warmer conditions indoors than outdoors generate relative increases in *R_0 _*(Table 2b). As differences between indoor and outdoor temperatures increase (e.g. with altitude), so does the relative change in transmission intensity. With the current data set, relative increases in *R_0 _*range from around 5 to approaching 3,000%, depending on the model and exact temperatures used.

## Implications of the hypothesis

Use of relative *R_0 _*does not in itself quantify absolute disease risk and as pointed out by Rogers & Randolph [4], even a large increase from a very small initial *R_0 _*will still be a small *R_0_*. Nonetheless, this analysis clearly reveals that current risk models based on mean outdoor temperatures could be substantially underestimating transmission intensity if mosquitoes spend significant periods of the gonotrophic cycle resting indoors. At higher altitudes, where the differences are greatest, indoor resting may be common as mosquitoes attempt to alleviate the burden of hostile outdoor microclimates [57]. Variation between individual villages indicates that application of a simple temperature correction factor is unlikely to capture the effects across different house structures and local environments.

The effect of the indoor microclimate will also extend to other temperature-dependent life-history characteristics, such as blood-meal digestion and egg production, potentially increasing malaria risk by endophilic mosquitoes even further. Understanding malaria transmission ecology and malaria risk, therefore, requires better awareness of mosquito resting behaviour and the associated microclimate. In this regard, the current study reveals a number of important knowledge gaps:

## Where do exophilic mosquitoes rest and what are the exact microclimates?

Searches for outdoor resting mosquitoes have frequently proved time consuming and unrewarding [58]. It seems likely that indoor collections will result in a relatively larger proportion of mosquitoes being caught, even when the outdoor resting population is of the same size or larger. A human dwelling is extremely small compared with its outdoor surroundings and so the size and importance of the exophilic fraction of a population is probably often overlooked [58,59]. The consequences of a prevailing sampling bias was highlighted recently with the discovery of a previously unknown subgroup of exophilic *An. gambiae *in Burkina Faso [60].

Outdoor resting mosquitoes seek shelter in a variety of environments, such as under the eaves of huts, in dry pots, canal water pipes, undersides of bridges, at bases of trees, in tree holes, piles of fallen leaves, cracks and crevices of brick pits, cracks and holes in the ground, small ridges under rocks, granaries, etc. [26,58,59,61,62]. All these sites are likely to be heavily shaded [61], and have their own specific microclimate. Outdoor temperature data, however, are most commonly collected from met-stations, such as Stevenson screens. Whether these met-station data are representative of the outdoor microclimates experienced by mosquitoes is unclear. Studies similar to the one by Meyer *et al *[63], in which microhabitat temperatures of resting places of *Culex tarsalis *were monitored in detail in California, USA, are urgently needed for tropical malaria mosquitoes.

## Where do endophilic mosquitoes rest and what are the exact microclimates?

Indoor temperature will strongly depend on factors such as season [14,41,42], location/altitude [14,40-42,50,54], the nature of the building structure [47,48,64], its surroundings [14,40-42], the number of occupants [44], and whether people burn wood indoors [65]. Additionally, even within a single house there is likely to exist a gradient of temperature microhabitats [66].

There appear few records of exactly where African malaria mosquitoes rest within a house. In Burkina Faso, 95% of the *An. gambiae *and *An. funestus *mosquitoes were resting on the ceiling [67]. In South-America, the preferred resting site within houses appears to differ between species and locations. In Columbia it was observed that *Anopheles darlingi *and *Anopheles marajoara *tended to rest close to the ground, whereas *Anopheles oswaldoi *and *Anopheles rangeli *rested higher up [68]. In Brazil, on the other hand, *An. darlingi *mosquitoes were mostly collected from the ceiling (59%), with 37% resting on the walls and only 4% on the floor [69]. In Guatemala, the greatest numbers (53%) of *Anopheles albimanus *were found at the undersurface of shed roofs, the remainder mostly on walls (28%) and furniture (13%) [70].

Similar variation has been observed in Asia for *Anopheles culicifacies*. In Maharashtra, India, about 70% were found resting on the underside of roofs of village houses while only 30% rested on the vertical walls and surface of furniture, vessels, grain bins, etc. In villages around Delhi, 72% of *An. culicifacies *were found resting on the ceiling and walls above 1.8 m from the floor [71]. In Sri Lanka, however, it was found that the species preferred to rest on walls below 1.8 m [72]. Whether such behavioural variation is important with respect to temperature, and is possibly even driven by microclimate selection, is uncertain.

## Do mosquitoes moderate body temperature via behavioural thermoregulation?

Short-term selection of thermally favoured microclimates, especially of sunny or shaded substrates, is probably the most common mechanism for control of body temperatures in insects [73]. The extent to which adult mosquitoes select habitats based on micro-environment and can behaviourally thermoregulate, however, is unclear. One study examined the escape or avoidance behaviour of *An. gambiae *and *An. arabiensis *in response to increasing temperatures and showed *An. arabiensis *to be slightly more thermally tolerant than *An. gambiae *[74]. Another study showed that newly-emerged *An. gambiae *mosquitoes can avoid desiccation by using their thermohygroreceptor cells to guide them to cooler and more humid locations that facilitate survival [75]. These observations are consistent with thermal gradient studies on *Anopheles stephensi*, which indicated some ability to avoid temperature extremes, although no clear capacity to maintain steady body temperatures by behavioural thermoregulation was observed [76]. Beyond these examples, research investigating whether adult mosquitoes make behavioural choices in response to temperature is limited. Given the potential importance of the behaviour, further studies, including whether malaria parasites have the potential to manipulate mosquito microhabitat selection (*cf*. [77]), would appear warranted.

## What are the implications of changes in resting behaviour due to vector control tools?

The use of insecticides on bed nets (ITNs) [78-81], eaves curtains [82], durable linings [83], or by indoor residual sprays (IRS) [84], all have the potential to keep/drive malaria vectors outdoors. Evolution of such behavioural resistance is a major threat to malaria control as all these front-line tools rely on mosquitoes feeding and/or resting indoors [85,86]. However, in some settings a switch to actual outdoor resting could result in increases in EIP and a reduction in underlying transmission intensity (Table 2). The extent to which such changes could offset any proximate reductions in control will depend on the specifics of the system. Based on the relative changes in *R_0 _*presented in Table 2, a shift to outdoor resting could reduce transmission intensity many fold and in theory, in areas where the outdoor environment it is too cold for parasites to complete their development within the lifespan of the mosquito, could even lead to local elimination.

The list of questions above highlights many unknowns and sets out numerous research challenges. Nonetheless, given that certain mosquitoes clearly spend part of the gonotrophic cycle indoors and that indoor microclimates do generally differ from outdoor microclimates, it seems important that malaria risk assessments move away from use of mean outdoor temperatures alone. Based on current knowledge it seems unlikely that processed-based models can fully capture the complexities of variable mosquito behaviours across variable environments. However, a useful starting point could be to take the extremes of complete exophily *vs *complete endophily and use these assumptions to explore the range of transmission intensity possible based on the full extent of microclimates available within a location.

## Competing interests

The authors declare that they have no competing interests.

## Authors' contributions

KPP and MBT both contributed to developing and writing the manuscript. Both authors read and approved the final manuscript.
